# Baby Killers: Documentation and Evolution of Scuttle Fly (Diptera: Phoridae) Parasitism of Ant (Hymenoptera: Formicidae) Brood

**DOI:** 10.3897/BDJ.5.e11277

**Published:** 2017-01-31

**Authors:** Brian V. Brown, John M. Hash, Emily A. Hartop, Wendy Porras, Dalton de Souza Amorim

**Affiliations:** 1 Natural History Museum of Los Angeles County, Los Angeles, United States of America; 2 University of California, Riverside, United States of America; 3 Universidade de São Paulo, Ribeirão Preto, Brazil

**Keywords:** tropical, parasitoids, rain forest, biodiversity

## Abstract

**Background:**

Numerous well-documented associations occur among species of scuttle flies (Diptera: Phoridae) and ants (Hymenoptera: Formicidae), but examples of brood parasitism are rare and the mechanisms of parasitism often remain unsubstantiated.

**New information:**

We present two video-documented examples of ant brood (larvae and pupae) parasitism by scuttle flies. In footage from Estação Biológica de Boracéia in Brazil, adult females of *Ceratoconus setipennis* Borgmeier can be seen attacking workers of *Linepithema humile* (Mayr) species group while they are carrying brood, and ovipositing directly onto brood in the nest. In another remarkable example, footage from the Soltis Center, near Peñas Blancas in Costa Rica, shows adult females of an unidentified species of the *Apocephalus grandipalpus* Borgmeier group mounting *Pheidole* Westwood brood upside-down and ovipositing while the brood are being transported by workers. Analysis of evolutionary relationships (in preparation) among *Apocephalus* Coquillett species shows that this is a newly derived behavior within the genus, as the *A. grandipalpus* group arises within a group of adult ant parasitoids. In contrast, relationships of *Ceratoconus* Borgmeier have not been studied, and the lifestyles of the other species in the genus are largely unknown.

## Introduction

The Phoridae (Insecta: Diptera) are a large group of small flies that are found worldwide. The ways of life of the majority of species are completely unknown, although they are frequently referred to as scavengers, based on the lifestyles of the best-known species. Enough fragmentary knowledge exists, however, to refer to phorids as one of the most biologically diverse groups of insects, with various species being scavengers, herbivores, fungivores, predators, parasitoids, and true parasites (compiled by Disney 1994).

As parasitoids (insects that feed on or in a single host for their development, eventually killing it), phorids have evolved to exploit a wide variety of hosts, but especially well-known is their use of ants (Formicidae), bees (mostly Apidae), millipedes (Diplopoda), and termites (Isoptera). Both commensal and parasitoid relationships with these other insects are common (with the exception of millipedes), but usually the parasitoids are confined to attacking adults of their hosts, as documented in copious field observations (e.g., Disney 1986, Brown 1997, Brown 2006, Feener 1981, Feener and Brown 1997, Porter 1998, Bragança et al. 1999, Hash and Brown 2015). Parasitism of ant larvae or pupae is a much rarer phenomenon; only a handful of examples of phorids reared from ant brood in the last century have been recorded (compiled by Disney 1994). These include species of *Apodicrania* Borgmeier parasitizing the larvae of the fire ant *Solenopsis saevissima* (Smith) (Williams and Whitcomb 1974), *Aenigmatias lubbocki* (Verrall) reared from *Formica transkaucasica* Nasonov pupae found in the nest (Donisthorpe 1927), and *Apocephalus* Coquillett sp. females observed landing on pupae being carried by *Pheidole dentata* (Smith) workers (LaBerge 1953).

In this paper we provide video documentation of startling newly observed behaviors in two species of phorid flies, showing that they are aggressive and unequivocal parasitoids of immature ants.

## Materials and Methods

In Brazil, ant nests were located by cutting open rotting logs in early November 2016. The exposed colonies were then observed to record phorid fly attack. Footage at the Estação Biológica de Boracéia was obtained with both an Olympus OMD-EM1 outfitted with a 60 mm Olympus macro lens (Figs 4, 6) and a Samsung Galaxy s6 Edge with a clip-on macro lens by Pocket Lens (Fig. 5). All specimens from Brazil were deposited in the collection of the Museu de Zoologia da Universidade de São Paulo.

**Figure 1.**
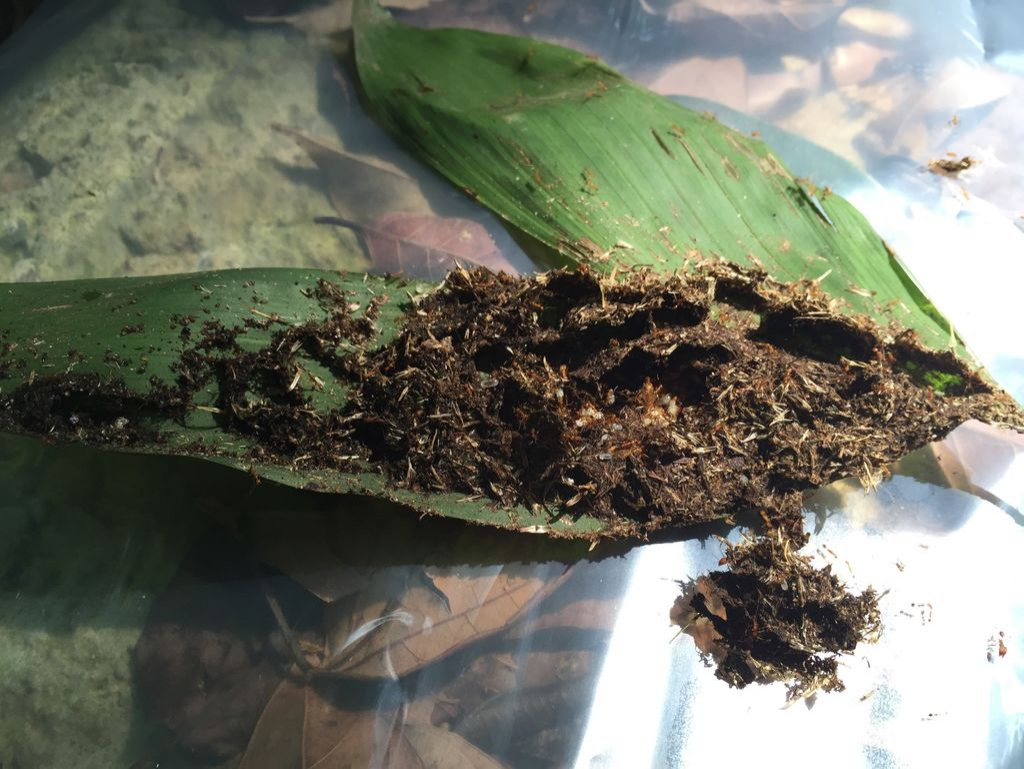
*Pheidole* sp nest on the underside of a *Chamaedorea* palm leaf.

In Costa Rica, *Pheidole* sp. nests were collected under the leaves of a *Chamaedorea* palm (Fig. 1) in late April 2016 and dissected, revealing the brood. Footage of these behaviors was obtained at the Texas A&M Soltis Center (10.383°N, 84.618°W, 468m) near Peñas Blancas, Alajuela, with an iPhone 6 camera with default hardware and software. Representative specimens of ants and phorid flies were collected into 95% EtOH and are currently in cold freezer storage at the University of California, Riverside.

Ants were identified by A. Wild. Phorids were identified by B. Brown, E. Hartop, and J. Hash. W. Porras and D. Amorim contributed to fieldwork.

## Results

**Figure 2.**
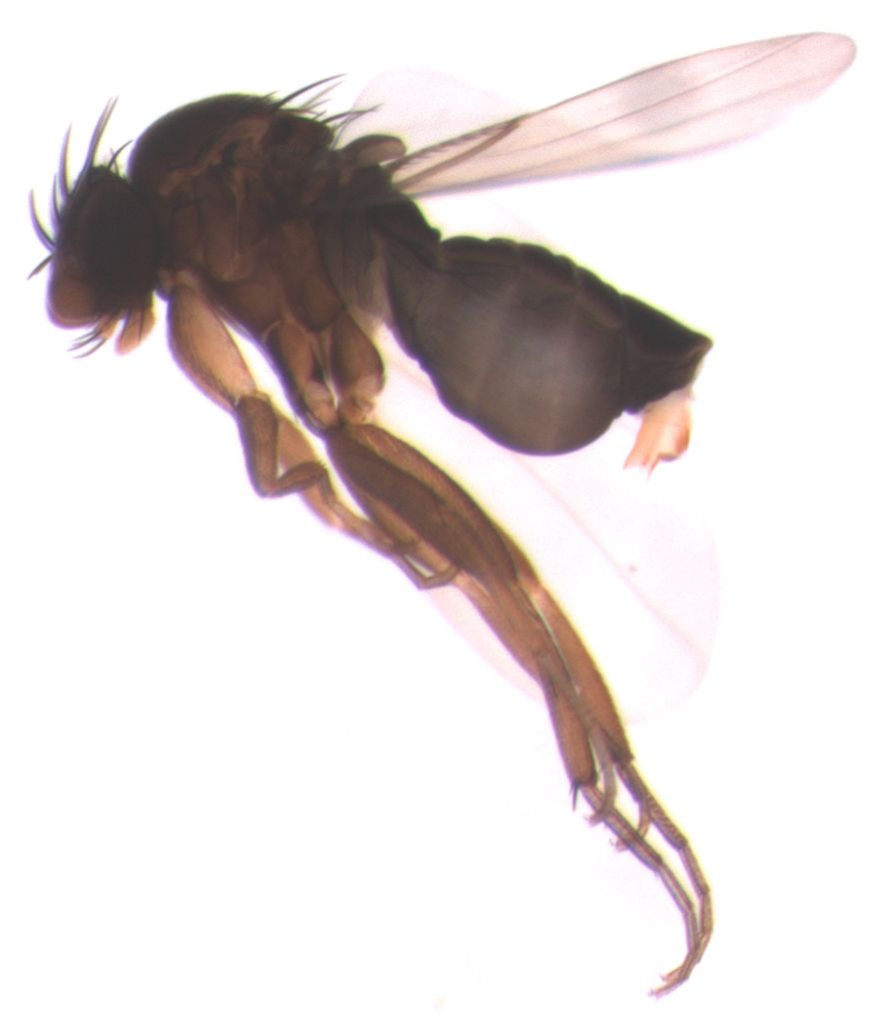
*Ceratoconus setipennis* female, lateral view.

**Figure 3.**
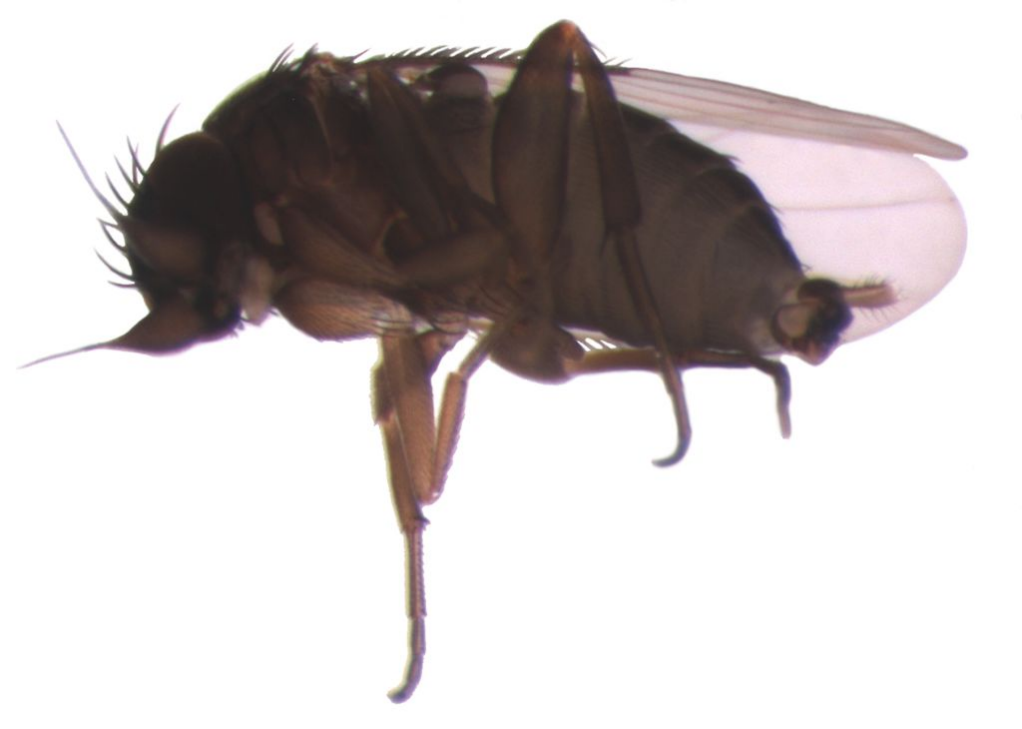
*Ceratoconus setipennis* male, lateral/ventral view.

Nests of the ant *Linepithema* sp were common in decaying logs in clearings and roadsides near the Boracéia station. Exposure of the colony quickly attracted both an aerial phorid parasitoid (*Pseudacteon* sp.) and individuals of *Ceratoconus setipennis* (Figs 2, 3). Adults of *C. setipennis* often arrived in copula, with the male quickly departing upon arrival at the host colony, as has been observed for several phorid parasitoids (Brown 2000, Brown et al. 2015). Upon colony exposure, ants worked quickly to remove exposed larvae and pupae to the inside of the nest (Fig. 4). Female flies conducted their attacks entirely on foot, following the brood-laden ants, and frequently “attacking” them by bumping into them, apparently in an attempt to induce the ants into dropping their loads (Fig. 5). On one occasion, a fly-pursued ant stashed a larva in a partly exposed position and then left it. This larva was attacked by the fly (Fig. 6). Flies also attacked and harassed worker ants carrying pupae, although no attempted ovipositions were witnessed.

The five known species of *Ceratoconus* are described only from Brazil (Borgmeier 1928, Prado 1976), but specimens from as far north as Costa Rica and as far south as Argentina are in the collection at the Natural History Museum of Los Angeles County. The genus was originally described as myrmecophilous, with the phorids living in symbiosis with the ants (based on the membranous ovipositor of the females). The ovipositor of *Ceratoconus setipennis* females is more membranous than that of phorids found to attack adult ants, but is noticeably modified from that of known commensals. Now that brood parasitism has been definitively recorded, this intermediate morphology is no longer a mystery. *Ceratoconus setipennis* was originally described as associated with *Linepithema humile* Mayr (formerly *Iridomyrmex humilis* Mayr). Specimens of *Pheidole rufipilis* Mayr were also present in the material examined by Borgmeier, but thought to have been mistakenly associated with the phorids (Borgmeier 1928). In our systematic destruction of the log where we made our observations, we encountered a number of ant species, but the phorids were only observed attacking the *Linepithema*.

In our Costa Rican example, *Pheidole* nests were generally restricted to single leaflets, often covering half or more of the under surface. In approximately half an hour of searching, 8-9 of these *Pheidole* nests were found. While dissecting nests on plastic sheets in the field, in search of *Pheidolomyia* Schmitz (Phoridae) females, large numbers of *Apocephalus* females arrived at the dissected nests. Female *Apocephalus grandipalpus* group females were observed running on the substrate and hovering above ants. Flies only briefly hovered above ants that were not carrying brood before moving back to the leaf substrate or assessing another ant (Fig. 7). Flies closely followed ants carrying brood for a longer duration until the ants moved under cover or until an "attack" commenced. We observed a striking instance of a hovering female approaching and attacking a large larva being carried by a worker. The oviposition behavior took place in less than two seconds. The female *Apocephalus* approached from behind the ant, landed on the larva and inserted her ovipositor; the ant pulled the larva and phorid under her body, while the phorid maintained contact with the larva before releasing and exiting under the body of the ant (Fig. 8).

## Discussion

The discovery of ant-brood parasitism in these two phorids demonstrates that this behavior has evolved at least twice in the family. The genus *Apocephalus* was proposed to be monophyletic by Brown (1997) based on characters not shared by *Ceratoconus*. Within *Apocephalus*, Brown also proposed several monophlyletic groups, including the large, still mostly unrevised, *Apocephalus grandipalpus* group. Although recent studies on relationships among these groups are not yet published, we have unpublished molecular information supporting the placement of the *A. grandipalpus* group well within the genus, clearly arising from adult-anti-parasitizing ancestors.

The relationships among *Ceratoconus* species, and of this genus to other phorid genera have not been studied. Some species are known, however, to be associated with army ants (Formicidae: Ecitoninae). These associations might be fortuitious, however, as army ant raids commonly induce colonies of ants to evacuate their nests, allowing phorid parasitoids access to the fleeing colony (Brown and Feener 1998). Indeed, species of the *Apocephalus grandipalpus* group are also frequently associated with army ant raids (B.Brown, unpublished observations). It is even possible that such raids made possible the switch in *Apocephalus* from adult ant parasitism to attacking brood, as mistaken ovipositions would be expected in the swirling chaos of an army ant raid. Unlike *Apocephalus*, which we frequently find associated with army ant raids, we have rarely, if ever, found species of *Ceratoconus* in this situtation; clearly, the relevance of army ant raids to *Ceratoconus* needs further study.

## Conclusions

The video documentation of two very different types of brood parasitism of ant species by scuttle flies was recorded in two countries within just a few months of one another. This hints at the many remarkable behaviors of phorid flies that may still await discovery by the patient observer. It appears brood parasitism may not be as rare as was once assumed, and that there may be a tremendous amount of information to uncover about these behaviors.

## Figures and Tables

**Figure 4.** Adult workers of *Linepithema* sp removing exposed larvae and pupae.

**Figure 5.** Female *Ceratoconus setipennis* attacking *Linepithema* sp carrying brood.

**Figure 6.** *Cerataconus setipennis* ovipositing on a *Linepithema* sp larva.

**Figure 7.** *Apocephalus* shown both ignoring *Pheidole* workers not carrying brood, and attacking a *Pheidole* larva as it is being transported by a worker.

**Figure 8.** The attack from Fig. 7 shown in slow motion.

## References

[B3476359] Borgmeier T. (1928). Investigações sobre phorideos myrmecophilos (Diptera, Phoridae). Archivos do Instituto Biologico.

[B3476482] Bragança M. A., Tonhasca A., Della Lucia T. M.C., Erthal M. (1999). Parasitismo de *Atta
sexdens* (Hymenoptera: Formicidae) por duas espécies de moscas da família Phoridae. Naturalia.

[B3477964] Brown BV (2000). Revision of the "*Apocephalus
miricauda*-group" of ant-parasitizing flies (Diptera: Phoridae). Contributions in Science.

[B3477954] Brown BV, Kung G, Porras W (2015). A new type of ant-decapitation in the Phoridae (Insecta: Diptera). Biodiversity Data Journal.

[B3476442] Brown B. V. (1997). Revision of the *Apocephalus* at*tophilus* group of ant-decapitating flies (Diptera: Phoridae). Contributions in Science.

[B3476432] Brown B. V. (2006). Revision of the untreated taxa of *Melaloncha* s. s. bee-killing flies (Diptera: Phoridae). Zootaxa.

[B3477994] Brown B V, Feener D H (1998). Parasitic phorid flies (Diptera: Phoridae) associated with army ants (Hymenoptera: Formicidae: Ecitoninae, Dorylinae) and their conservation biology. Biotropica.

[B3476541] Disney R. H.L. (1986). Two remarkable new species of scuttle-fly (Diptera: Phoridae) that parasitize termites (Isoptera) in Sulawesi. Systematic Entomology.

[B3476331] Disney R. H. L. (1994). Scuttle Flies: The Phoridae.

[B3476512] Donisthorpe H. (1927). The Guests of British Ants: Their Habits and Life Histories.

[B3476462] Feener D. H. (1981). Competition between ant species: outcome controlled by parasitic flies. Science.

[B3476452] Feener D. H., Brown B. V. (1997). Diptera as parasitoids. Annual Review of Entomology.

[B3477984] Hash JM, Brown BV (2015). Revision of the New World species of the millipede-parasitic genus *Myriophora* Brown (Diptera: Phoridae). Zootaxa.

[B3476521] LaBerge W. E. (1953). A note on a phorid parasite of *Pheidole
dentata* Mayr. Journal of the Kansas Entomological Society.

[B3476531] Porter S. D. (1998). Host-specific attraction of *Pseudacteon* flies (Diptera: Phoridae) to fire ant colonies in Brazil. Florida Entomologist.

[B3476369] Prado A. (1976). Records and descriptions of phorid flies, mainly of the Neotropical Region (Diptera: Phoridae). Studia Entomologia.

[B3476492] Williams R. N., Whitcomb W. H. (1974). Parasites of fire ants in South America. Proceedings of the Tall Timbers Conference on the Ecology of Animals and its Contribution to Habitat Management.

